# Evaluation parameters of graft maturation on second-look arthroscopy following anterior cruciate ligament reconstruction: a systematic review

**DOI:** 10.1186/s43019-019-0005-3

**Published:** 2019-06-28

**Authors:** Sang-Gyun Kim, Jae Hyun Jung, Jong-Hyub Song, Ji-Hoon Bae

**Affiliations:** 1 Department of Orthopaedic Surgery, Korea University Guro Hospital, Korea University College of Medicine, 148 Gurodong-ro, Guro-gu, Seoul, 08308 Republic of Korea; 2 Department of Rheumatology, Korea University Guro Hospital, Korea University College of Medicine, Seoul, Republic of Korea

**Keywords:** Knee, Anterior cruciate ligament reconstruction, Second-look surgery, Graft, Maturation, Systematic review

## Abstract

**Purpose:**

The purpose of this systematic review was to investigate and summarize the evaluation methods of graft maturation on second-look arthroscopy following anterior cruciate ligament (ACL) reconstruction.

**Methods:**

A literature search was performed on articles before December 2017 to identify the literature that has evaluated graft maturation on second-look arthroscopy following ACL reconstruction. Only studies using human grafts, evaluating graft maturation with two or more gross findings were included. Study design, grafts, surgical techniques, follow-up period, evaluation parameters, and categories were compiled.

**Results:**

Twenty-eight studies were included in this study. All studies evaluated graft maturation with two or more of the following three findings: graft integrity, tension, and synovial coverage. Two to four categories were used for evaluating each parameter, but the criteria for classification were slightly different for each study. Several studies reported neo-vascularization of grafts and the total maturation score by summing up the scores assigned to each evaluation parameter. Three studies reported that there was no correlation between second-look findings and patient-reported outcomes.

**Conclusions:**

Graft integrity, tension, and synovial coverage were the most frequently evaluated for graft maturation on second-look arthroscopy. However, there is no uniform criterion for evaluation. Therefore, development of a valid, uinform criterion is required.

**Level of evidence:**

Level IV, systematic review of level I–IV investigations.

## Introduction

After anterior cruciate ligament (ACL) reconstruction, the graft tendon undergoes a maturation process called “ligamentization”. Amiel et al. [1] described this phenomenon as the continuous development of a tissue, which was a tendon originally, into a substance very similar to a normal ACL. For long-term survival of grafts, favorable biologic quality with good ligamentization as well as mechanical properties would be essential [2, 3]. Recent studies have shown that autograft is superior to allograft in long-term survival [2, 4], which may be different in biologic maturation.

Many studies have evaluated graft maturation using second-look arthroscopy [5–9]. This method has several disadvantages. Histologic findings cannot be confirmed and the maturation of the graft depends on the subjective evaluation of the surgeon. However, second-look arthroscopy has several advantages over biopsy. It does not cause damage to the grafts by evaluating the graft maturation indirectly. Also, it doesn’t result in different outcomes depending on the site from where the tissue is taken. For these reasons, many studies have evaluated graft maturation with gross findings seen in the second-look arthroscopy until recently [10–14].

However, there was no consensus on which parameters should be evaluated and which criteria should be used for each parameter to evaluate graft maturation on second-look arthroscopy. Surgeons have been using their own criteria for evaluating graft maturation. Toritsuka et al. [5] evaluated graft maturation by classifying the integrity and tension of the graft observed on the second-look arthroscopy into four categories, whereas Ahn et al. [6] and Kondo et al. [7] evaluated it by classifying the integrity, tension, and synovial coverage of grafts into three categories.

The purpose of this study was to review the previous literature (1) to investigate how to evaluate graft maturation on second-look arthroscopy following ACL reconstruction and (2) to determine if second-look arthroscopy had an objective evaluation value. We hypothesized there would be a lot of different methods to perform second-look arthroscopy following ACL reconstruction and second-look findings would be less correlated to the clinical outcomes.

## Methods

### Study eligibility criteria

We performed a systematic review of all the literature on second-look arthroscopy following ACL reconstruction, regardless of the level of study, graft type, and surgical technique (femoral tunnel placement, single bundle or double bundle reconstruction). Searches were restricted to papers published in English. Only articles with detailed description on how to evaluate graft maturation were included. The mean follow-up period of all included studies was more than one year.

### Literature search

Our literature search consisted of searches in PubMed and Embase for the terms “anterior cruciate ligament”, “reconstruction”, and “second-look” from the inception of these search engines till December 2017. After removing duplications, we checked the titles and abstracts of all articles to determine whether they fit the previously established inclusion criteria. The included articles identified by the search were each analyzed by a senior author to ensure their appropriateness.

**Fig. 1:**
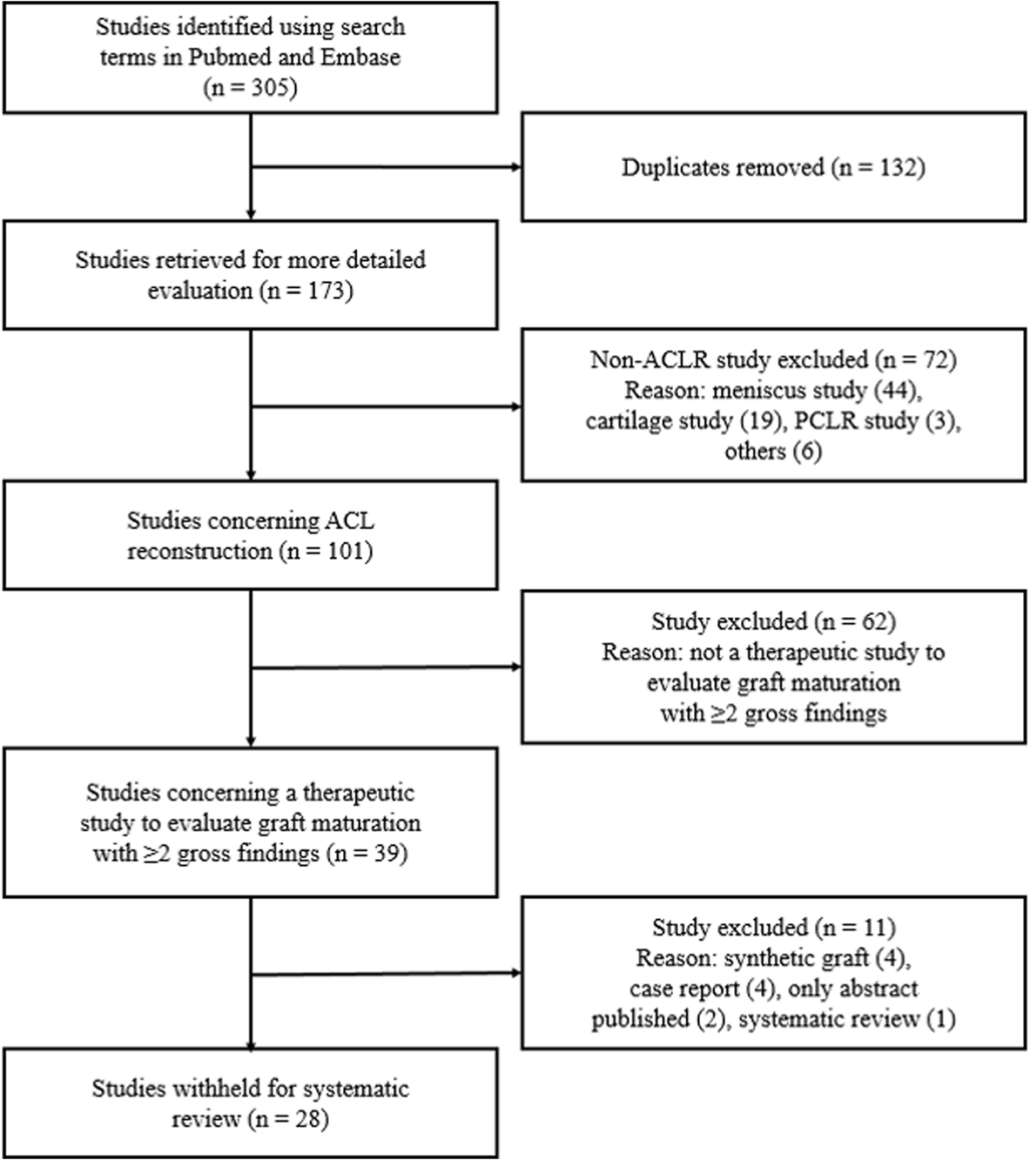
Quality of Reporting of Meta-analyses (QUOROM) flow diagram depicting the number of studies identified, included, and excluded as well as the reasons for exclusion

Only human ACL reconstruction studies using human grafts and evaluating graft maturation on second-look arthroscopy with two or more gross findings were included. As it was difficult to comprehensively evaluate graft maturation with only one parameter, we included only those studies that evaluated two or more gross findings of the grafts. In addition, studies with synthetic grafts, case reports, studies for which only abstracts had been published without full text, and systematic review studies were excluded. The results of this literature review are outlined in the Preferred Reporting Items for Systematic Reviews and Meta-Analyses (PRISMA) diagram in Fig. 1.

### Data extraction

The data from each of the 28 articles meeting the inclusion criteria for our systematic review were compiled. We collected demographic data, such as the graft type, number of second-look arthroscopies performed, and follow-up period from reconstruction to second-look arthroscopy. The reconstruction techniques (surgical techniques for femoral tunnel placement, single bundle or double bundle reconstruction) were recorded from all studies that reported them. We investigated the parameters used for evaluating second-look arthroscopy and categories that were used for each parameter to evaluate graft maturation in those studies.

### Study quality assessment

To assess the methodological quality, the modified Coleman Methodology Scores (mCMS) and subscales were determined for each included study [15]. The final score ranges from 0 to 100, with a score of 100 indicating the highest study quality. In addition, all included studies were assessed for level of evidence according to the Oxford Centre for Evidence-Based Medicine [16]. Most studies had retrospective, non-randomized designs. There were only five randomized controlled trials or prospective cohort studies (levels of evidence I and II) [7, 8, 14, 17, 18], probably because of the invasive nature of second-look arthroscopy. Eleven of the remaining 23 studies were retrospective comparative studies (level of evidence III) and 12 studies were case series (level of evidence IV).

## Results

An overview of the 28 studies showing year and journal of publication, level of evidence, graft type, number of second-look arthroscopies performed, reconstruction technique used, and follow-up period from ACL reconstruction to second-look arthroscopy is provided in Table 1. The parameters and categories for evaluating graft maturation on second-look arthroscopy are summarized in Table 2.

**Table 1:** Selected study characteristics

	Year of publication (journal)	Level of evidence	Mean age of patients (year)	Grafts (number of cases)	Technique	Follow-up (months)	mCMS
Defrere et al. [19]	1994 (*CORR*)	IV	32 (18.9–70.8)	FL: 47	TT, SB	1 year after surgery	41
Jung et al. [20]	1997 (*Bull Hosp Jt Dis*)	IV	28 (20–38)	BT-AT: 19	Not described, SB	BT-AT: 15.8 (12–28)	39
Toritsuka et al. [5]	2004 (*Arthroscopy*)	IV	24 (15–51)	HA: 156	Not described, SB/DB	< 12, 12–24, > 24 (5–50)	49
Iwai et al. [21]	2005 (*AOTS*)	III	24.1 (15–44)	BT-AT + ITB: 43 BT-AT alone: 45	Not described, SB	> 24	46
Ahn et al. [6]	2007 (*Arthroscopy*)	IV	29.1 (15–54)	HA: 74	TT, SB	20.1 (9–32)	43
Ahn et al. [22]	2007 (*KSSTA*)	III	31 (16–60)	BT-AT: 80 HA: 129	TT, SB	21.2 (14–70)	49
Kondo et al. [7]	2007 (*Arthroscopy*)	II	27 (13–62)	HA: 136	TT, DB	14 (11–24)	59
Otsubo et al. [23]	2007 (*KSSTA*)	IV	24.1 (15–44)	HA: 65	TP, DB	16.5 (5–29)	43
Lee et al. [24]	2010 (*Arthroscopy*)	IV	BT-AL: 27.9 (13–60)	BT-AL: 16	TT, SB	BTB: 38 (17–69)	46
			TA: 28.6 (15–60)	TA: 47		TA: 33 (12–66)	
			HA: 29.3 (15–52)	HA: 43		HA: 27 (15–50)	
Mae et al. [25]	2010 (*Arthroscopy*)	IV	31.2 (15–52)	HA: 25	OI, DB	13.9 (10–21)	39
Ahn et al. [26]	2011 (*JBJS*)	IV	31 (15–58)	HA: 37	TT, DB	> 24	39
Ahn et al. [27]	2011 (*Arthroscopy*)	IV	32.2 (17–54)	HA: 33	TT, SB	> 12	39
Kinugasa et al. [8]	2011 (*Arthroscopy*)	II	30.9 (14–71)	HA (A): 55	OI, DB	HA (A): 14.3 (11–20)	56
				HA (B): 36		HA (B): 13.1 (8–20)	
				HA (C): 11		HA (C): 15.5 (12–20)	
Ohsawa et al. [28]	2012 (*AJSM*)	III	AM: 24.9 (15–50)	HA: 99	TP/OI, DB	1 year after surgery	46
			PL: 24.1 (14–46)				
Ohsawa et al. [29]	2012 (*Arthroscopy*)	IV	36.8 (15–57)	HA: 19	TP, selective SB	1 year after surgery	39
Tanaka et al. [30]	2012 (*KSSTA*)	IV	25.5 ± 8.5	HA: 41	TP, TB	6–22	43
Kim et al. [31]	2014 (*Knee*)	III	RP: 28.9 ± 8.8	HA (RP): 36	TT, SB	HA (RP): 27.5 ± 3.1	43
			RS: 32.0 ± 9.4	HA (RS): 30		HA (RS): 26.6 ± 2.1	
Nakamae et al. [32]	2014 (*BJJ*)	III	SB: 24.6 ± 11.9	HA (SB): 61	Not described, SB/DB/AG	HA (SB): 25.6 (18–72)	53
			DB: 24.8 ± 11.0	HA (DB): 82			
			AG: 26.6 ± 11.4	HA (AG): 73		HA (DB): 24.2 (18–41)	
						HA (AG): 23.1 (18–72)	
Ahn et al. [9]	2015 (*Arthroscopy*)	III	PA: 37.2 (19–61)	HA (PA): 88	TP, DB	HA (PA): 45.0 (25–77)	52
			NA: 34.7 (15–57)	HA (NA): 66		HA (NA): 45.5 (25–87)	
Kondo et al. [17]	2015 (*AJSM*)	II	29 (13–58)	HA (RP): 62	TT, DB	14 (11–24)	56
				HA (RS): 46			
Lu et al. [18]	2015 (*AJSM*)	II	26.3 (18–32)	HA (EF): 28	TP, DB	HA (EF): 80.6 ± 16.3	61
						HA (BL): 65.2 ± 12.8	
				HA (BL): 31			
Choi et al. [33]	2017 (*Knee*)	III	29.1 (15–54)	HA/ACH (RP): 61	TT, SB	HA/ACH (RP): 14.8 (10–23)	46
				HA/ACH (RS): 30		HA/ACH (RS): 15.1 (10–25)	
Kim et al. [10]	2017 (*KSSTA*)	III	RP: 30.3 ± 11.6	QA (RP): 42	TT, SB	QA (RP): 17.9 ± 6.4	43
			RS: 26.3 ± 8.4	QA (RS): 33		QA (RS): 18.2 ± 5.8	
Kim et al. [11]	2017 (*KSSTA*)	III	TA: 30.8 ± 13.3	TA: 30	TP/OI, SB	TA: 22.5	47
			HA: 28.9 ± 10.1	HA: 26		HA: 22.5	
Matsushita et al. [12]	2017 (*KSSTA*)	IV	PL: 35.1 ± 16.4	HA (PL): 16	TP, SB/DB	1 year after surgery	43
			SB: 30.1 ± 10.5	HA (DB): 37			
Nakayama et al. [13]	2017 (*Knee*)	III	PR: 26.6 (14–55)	HA (PR): 14	OI, DB	1 year after surgery	39
			RS: 26.4 (12–59)	HA (RS): 22			
Xu et al. [34]	2017 (*Med Sci Monit*)	III	HA: 32.8 ± 8.9	HA: 31	TP, SB	HA: 28.4 ± 2.1	43
			HY: 33.9 ± 8.4	HY: 37		HY: 28.3 ± 2.8	
Yoo et al. [14]	2017 (*KSSTA*)	I	TA: 24 (13–52)	TA: 25	TP, SB	TA: 34.5 (25.3–59.5)	58
			HA: 30 (15–62)	HA: 26		HA: 32.8 (28.7–50.1)	

*mCMS* modified Coleman Methodology Scores, *FL* fascia lata allograft, *BT-AT* bone patella bone tendon autograft, *BT-AL* bone patella bone tendon allograft, *TA* tibialis allograft, *HA* hamstring autograft, *HY* hybrid graft, *QA* quadriceps autograft, *ACH* Achilles allograft, *TT* transtibial technique, *TP* transportal technique, *OI* outside-in technique, *SB* single bundle reconstruction, *AG* single bundle augmentation, *DB* double bundle reconstruction, *TB* triple bundle reconstruction, *PL* posterolateral bundle reconstruction, *RP* remnant preservation, *RS* remnant sacrifice, *PA* provisional anatomic reconstruction, *NA* non-anatomical reconstruction, *AM* anteromedial, *PL* posterolateral

*CORR* Clinical Orthopaedics and Related Research, *Bull Hosp Jt Dis* Bulletin of the Hospital for Joint Diseases, *AOTS* Archives of Orthopaedic and Trauma Surgery, *KSSTA* Knee Surgery, Sports Traumatology, Arthroscopy, *JBJS* Journal of Bone & Joint Surgery, *AJSM* The American Journal of Sports Medicine, *BJJ* The Bone & Joint Journal, *Med Sci Monit* Medical Science Monitor

**Table 2:** Summary of parameters and categories used for evaluating graft maturation on second-look arthroscopy

	Integrity	Tension	Synovial coverage
Defrere et al. [19] (*CORR*, 1994)	1. Invested either by a synovial layer or by neovascularization		
	- Perfect stability; slight instability		
	2. Non-invested (without sign of above)		
	- Perfect stability; slight instability; rupture in one half; graft failure		
Jung YB et al. [20] (*Bull Hosp Jt Dis*, 1997)	One or more of the following findings:		
	Nearly normal appearance; incomplete synovial coverage; partially torn fibers at the femoral tunnel site;		
	parallel fragmentation with cyclops lesion; impingement without the damage of the ACL graft		
Toritsuka et al. [5] (*AOTS*, 2005)	No tear;	Arthroscopic probe test	NA
	Partial tear	Taut (as normal ACL);	
	- Superficial partial tear	Mildly lax (less tension,	
	- Substantial partial tear	showing redundancy);	
	- Partial tear, not classified	Lax (obvious loss of tension)	
Iwai et al. [21] (*AOTS*, 2005)	Fibrous split (+)	Arthroscopic probe test	NA
	Fibrous split (−)	Normal tension;	
		Decreased tension	
Ahn et al. [6] (*Arthroscopy*, 2007)	Normal;	Arthroscopic probe test	Good (complete)
	Partial tear	Taut; slightly lax	Half (insufficient)
	Complete tear		Pale (severely insufficient)
Ahn et al. [22] (*KSSTA*, 2007)	Normal;	Arthroscopic probe test	Fair (well covered)
	Partial tear;	Taut; slightly lax	Poor (barely covered)
	Complete tear		
Kondo et al. [7] (*Arthroscopy*, 2007)	A (no laceration or elongation of thick graft)		A (completely covered)
	B (partial laceration of thick graft or no laceration or elongation of thin graft)		B (partial)
	C (complete tear or obvious elongation)		C (almost not)
Otsubo et al. [23] (*KSSTA*, 2007)	No rupture	Arthroscopic probe test	Good (whole length)
	Partial rupture	Taut (normal ACL)	Fair (> 50%)
	Total rupture	Lax (loss of tension)	Poor (< 50%)
Lee et al. [24] (*Arthroscopy*, 2010)	Normal	Arthroscopic probe test	Normal (> 50%)
	Abnormal (tear or cyclops lesion)	Normal (< 3 mm)	Abnormal (< 50%)
		Nearly normal (3–5 mm)	
		Abnormal (5–10 mm)	
		Severely abnormal (> 10 mm)	
Mae et al. [25] (*Arthroscopy*, 2010)	No tear;	Arthroscopic probe test	Good (whole length)
	Superficial tear	Taut; mildly lax; lax	Fair (> 50%)
			Poor (< 50%)
	Substantial tear		
Ahn et al. [26] (*JBJS*, 2011)	A (no laceration or elongation of thick graft)		A (complete)
	B (partial laceration of thick graft or no laceration/elongation of thin graft)		B (partial)
	C (complete tear or obvious elongation)		C (almost not)
Ahn et al. [27] (*Arthroscopy*, 2011)	No tear	Arthroscopic probe test	Good
	Partial tear	Taut; mildly lax; lax	Fair
	Complete tear		Poor
Kinugasa et al. [8] (*Arthroscopy*, 2011)	No tear	Arthroscopic probe test	Good (> 80%)
	Superficial tear	Taut; mildly lax; lax	Fair (50–80%)
	Substantial tear		Poor (< 50%)
Ohsawa et al. [28] (*AJSM*, 2012)	No tear (> 80%)	Arthroscopic probe test	Excellent (entire coverage)
	Partial tear (30–80%)	Taut (< 3 mm, firm end point)	Fair (partial defect)
	Complete tear (< 30%)	Slightly lax (> 3 mm, firm end point)	Poor (barely covered)
		Lax (no firm end point)	
Ohsawa et al. [29] (*Arthroscopy*, 2012)	No tear (> 80%)	Arthroscopic probe test	Excellent (entire coverage)
	Partial tear (30–80%)	Taut (< 3 mm, firm end point)	Fair (partial defect)
	Complete tear (< 30%)	Slightly lax (> 3 mm, firm end point)	Poor (barely covered)
		Lax (no firm end point)	
Tanaka et al. [30] (*KSSTA*, 2012)	No tear	Arthroscopic probe test	Good (whole length covered)
	Substantial tear	Taut (as normal ACL)	Fair (> 50%)
		Lax (looser than normal)	Poor (< 50%)
Kim et al. [31] (*Knee*, 2014)	No tear	No arthroscopic test	Good
	Partial tear	(tested by KT-2000 and PST)	Partial
			Poor
	Complete tear		
Nakamae et al. [32] (*BJJ*, 2014)	Normal		1. Synovial coverage of graft:
	Damaged (obvious lack of tension or a substantial tear)		good (> 80%); fair (50–80%); poor (< 50%)
			2. Synovial coverage between the graft and the femoral bone tunnel:
			good (> 80%); poor (< 80%)
Ahn et al. [9]^†^ (*Arthroscopy*, 2015)	≥ 90%; 75–90%; 50–75%; < 50%	Arthroscopic probe test	≥ 75%; 50–75%; 25–50%; < 25%
		< 2 mm; 2–5 mm; 5–10 mm; > 10 mm	
Kondo et al. [17] (*AJSM*, 2015)	I (no laceration or tear)	No arthroscopic test	I (completely with thick tissue)
	II (partial laceration)	(tested by KT-2000 and PST)	II (completely with thin tissue)
			III (partly with thin tissue or almost not covered)
	III (complete tear or obvious elongation)		
Lu et al. [18]^†^ (*AJSM*, 2015)	No tear (> 80%)	Arthroscopic probe test	Synovial coverage and vascularization
	Partial tear (30–80%)	Taut (tense as normal ACL)	Excellent (entirely covered)
	Complete tear (< 30%)	Mildly lax (less tension, redundant)	Fair (partial defect)
		Lax (obvious loss of tension)	Poor (barely covered)
Choi et al. [33] (*Knee*, 2017)	N/A	Arthroscopic probe test and	Excellent (> 80%)
		intra-operative anterior drawer test	Good (50–80%)
		(translation was measured by ruler)	Fair (20–50%)
			Poor (< 20%)
Kim et al. [10] (*KSSTA*, 2017)	I (> 80% intact)	Arthroscopic probe test	I (> 80%)
	II (< 80% intact)	I (< 3 mm, firm end)	II (30–80%)
	III (< 30% intact)	II (> 3 mm, firm end);	III (< 30%)
		III (no firm end)	
Kim et al. [11]^†^ (*KSSTA*, 2017)	Complete tear	No arthroscopic test	> 75%
	Partial tear	(tested by Lachman test and	25–75%
	No tear	stress radiograph)	< 25%
Matsushita et al. [12] (*KSSTA*, 2017)	No laceration	No elongation	Completely covered
	Partial tear	Elongation of thin graft	Partially covered
	Complete tear	Obvious elongation	Almost not covered
Nakayama et al. [13] (*Knee*, 2017)	Good (no partial tear); fair (superficial tear); poor (substantial tear)	No arthroscopic test (tested by KT-1000)	Good (complete); fair (> 50%); poor (< 50%)
Xu et al. [34] (*Med Sci Monit*, 2017)	Good	Arthroscopic probe test	> 75%; 50–75%; 25–50%; < 25%
	Fair	Taut; mildly lax; lax	
	Damaged (re-tear)		
Yoo et al. [14] (*KSSTA*, 2017)	Intact	No arthroscopic test	Good (> 20%)
	Partial tear; total tear	(tested by Lachman test and PST)	Poor (< 20%)

†Three studies also evaluated neo-vascularization of grafts. *PST* pivot-shift test

*CORR* Clinical Orthopaedics and Related Research, *Bull Hosp Jt Dis* Bulletin of the Hospital for Joint Diseases, *AOTS* Archives of Orthopaedic and Trauma Surgery, *KSSTA* Knee Surgery, Sports Traumatology, Arthroscopy, *JBJS* Journal of Bone & Joint Surgery, *AJSM* The American Journal of Sports Medicine, *BJJ* The Bone & Joint Journal, *Med Sci Monit* Medical Science Monitor

### Study identification and characteristics

The literature review described above yielded 28 manuscripts that met all inclusion criteria (see PRISMA flow diagram in Fig. 1). All studies except one [19] were from northeast Asian regions including South Korea [6, 9–11, 14, 20, 22, 24, 26, 27, 31, 33], Japan [5, 7, 8, 12, 13, 17, 21, 23, 25, 28–30, 32], and China [18, 34]. Year of publication ranged from 1994 to 2017. Various grafts (autograft, allograft) were used and the mean follow-up period from reconstruction to second-look arthroscopy was more than 1 year. Regarding reconstruction technique, anatomical and non-anatomical reconstructions and single bundle and double bundle reconstructions were all included (there was one triple bundle reconstruction by Tanaka et al. [30]). All studies evaluated graft maturation with two or more of the following three gross findings: graft integrity, tension, and synovial coverage. Most of the studies had evaluated each parameter separately, while some papers evaluated two or more parameters together [7, 19, 20]. The mean mCMS was 46.4 ± 6.7 (range 39 to 61). The corresponding values for each study are shown in Table 1.

### Graft integrity on second-look arthroscopy

Most studies (27/28) evaluated the integrity of the graft on second-look arthroscopy. The most common classification of graft integrity involved three categories according to the severity of graft tear: intact, partial (or superficial) tear, and complete (or substantial) tear [13, 25, 31]. Moreover, five studies categorized the percentage of the intact portion of the entire graft volume [9, 10, 18, 28, 29]. Ohsawa et al. [28, 29] described the integrity of the graft as “no tear” when more than 80% of the graft was intact, “partial tear” when 30–80% of the graft was intact, and “complete tear” when less than 30% of the graft was intact. Subsequently, other studies also used these criteria to assess the integrity of the graft [10, 18]. On the other hand, Ahn et al. [9] classified graft integrity in four categories according to their own criteria (≥ 90%, 75–90%, 50–75%, < 50%).

### Graft tension on second-look arthroscopy

Twenty-two of the total studies evaluated the tension of the graft by probing during second-look arthroscopy. In addition, most studies objectively evaluated the graft tension using physical examination, Telos stress radiography, and KT-arthrometer. The most common classification of graft tension also involved three categories depending on how the graft moved during probing: taut, slightly (mildly) lax, and lax. Nine studies used these categories [5, 8, 10, 18, 25, 27–29, 34]. “Taut” is the same tension as normal ACL when moving within 3 mm of tension or probing. “Slightly lax” is laxer than normal ACL, moving 3 mm or more when probing but with a firm endpoint felt. Finally, “lax” is used in cases where the tension of the graft is not felt and on probing moves 10 mm or more. Five studies classified graft tension into two categories (normal and abnormal) [6, 21–23, 30], whereas the other two studies classified it into four categories according to their own criteria (< 2 or 3 mm, 2 or 3 mm to 5 mm, 5 to 10 mm, > 10 mm) [9, 24].

### Synovial coverage of graft on second-look arthroscopy

Most studies (26/28) used synovial coverage to evaluate graft maturation in second-look arthroscopy. The most common classification of synovial coverage involved three categories. However, the criteria for assigning the categories vary by study. Kondo et al. [7] classified graft maturation as completely covered, partially covered, and almost not covered according to synovial coverage of the graft. Kinugasa et al. [8] classified it according to the synovial coverage percentage—more than 80% as good, less than 50% as poor, and intermediate as fair—while Kim et al. [11] used values of 75%, 25–75%, and 25%, respectively. Among the studies that classified synovial coverage into two categories, Otsubo et al. [23] referred to more than 50% of synovial coverage as normal, but Yoo et al. [14] considered more than 20% of synovial coverage as good. Some studies used two [14, 22, 24] or four categories [9, 33, 34] to evaluate the synovial coverage of the graft.

### Neo-vascularization on second-look arthroscopy

Only three of the included studies used neo-vascularization for evaluating graft maturation [9, 11, 18]. Ahn et al. [9] classified synovial coverage with re-vascularization and assigned scores as follows: synovial coverage ≥ 75% and abundant re-vascularization (4 points); synovial coverage ≥ 75% but lack of revascularization (3 points); synovial coverage of 50–75% (2 points); synovial coverage of 25–50% (1 point); and synovial coverage < 25% (0 points). Lu et al. also combined synovial coverage and vascularization classifying into 3 categories [18]. Kim et al. [11] evaluated the vascular coverage of grafts using values of > 75%, 25–75%, and < 25%.

### Total graft maturation score and grading system

Seven studies tried to evaluate graft maturation comprehensively by combining various parameters. They calculated the total graft maturation score by summing up the scores assigned to each evaluation parameter. Details of each study are summarized in Table 3. The Kondo and Yasuda were the first to use the total graft maturation score and grading system [7], and several studies have used this method to evaluate the maturation of grafts [12, 17]. Ahn et al. [9] evaluated three parameters, graft integrity, tension, and synovial coverage with revascularization. They compared the graft maturation score and clinical outcomes between the anatomic reconstruction group and non-anatomic reconstruction group. Kim et al. [11] also used total graft maturation score to compare graft maturation between hamstring autografts and tibialis allografts. However, only one of the seven studies investigated the correlation between total maturation score and clinical outcome [7].

**Table 3:** Summary of the Studies that Used Total Maturation Score or Grading System

	Method	Correlation with clinical outcomes
Kondo et al. [7] (*Arthroscopy*, 2007)	Graft maturation score for each bundle based on graft tension (0–2)* and integrity (0–2). Each bundle (AM, PL) with a total score of 4 was evaluated as excellent, 2 or 3 as fair, and 0 or 1 as poor. DB grafts were classified into categories I (two excellent bundles), II (only one excellent bundle), or III (no excellent bundle)	Category I showed significantly better results than categories II or III in KT-2000 and PST
		No significant differences between the three categories in all clinical outcomes.
Ahn et al. [9] (*Arthroscopy*, 2015)	Graft maturation score based on integrity (0–3), tension (0–3), and synovial coverage with neo-vascularization (0–4). Total graft maturation scores ranged from 0 to 10 points	No comparison between second-look findings and objective outcomes, but remnant preserved group showed higher graft maturation score and better clinical outcomes
Kondo et al. [17] (*AJSM*, 2015)	Graft maturation score and grade system by Kondo and Yasuda were used	No comparison between second-look findings and objective outcomes, but remnant preserved group showed better maturation grade and stability (KT-2000, PST)
Lu et al. [18] (*AJSM*, 2015)	Graft maturation score based on synovial and vascular coverage, tension, integrity. A maximum of 2 points was assigned for each parameter. A graft with a total score of 5 or 6 was evaluated as excellent, 3 or 4 as fair, and ≤ 2 as poor	No comparison between second-look findings and objective clinical outcomes. But ACLR using the existing footprint remnant for tunnel placement showed higher graft maturation score and better functional results (ROM recovery, subjective outcome scores)
Kim et al. [10] (*KSSTA*, 2017)	Total maturation score based on graft tension, integrity, and synovial coverage (modified from method by Ohsawa et al. [28]). According to the second-look finding, a maximum of 3 points was assigned for each parameter. Total scores ranged from 3 to 9 points	No comparison between total maturation score and clinical outcomes
Kim et al. [11] (*KSSTA*, 2017)	Total maturation score based on graft tension, integrity, synovial coverage, and revascularization. According to the second-look finding, a maximum of 2 points was assigned for each parameter. Total scores ranged from 0 to 8 points	No comparison between total maturation score and clinical outcomes
Matsushita et al. [12] (*KSSTA*, 2017)	Graft maturation grade and category system by Kondo and Yasuda [17]	No comparison between total maturation grade/category and clinical outcomes

*Assigned score for each parameter in blank. *AM* anteromedial bindle, *PL* posterolateral bundle, *DB* double bundle, *ACLR* anterior cruciate ligament reconstruction

*AJSM* The American Journal of Sports Medicine, *KSSTA* Knee Surgery, Sports Traumatology, Arthroscopy

### Correlation between the second-look findings and clinical outcomes

Nine of the included studies compared second-look findings with clinical outcomes (Table 4). Most studies (8/9) compared the results of the second-look findings and objective stability (KT-arthrometer, physical examination). With regard to stability, two studies reported that graft tension in the second-look arthroscopy was correlated with objective stability [5, 22], but this was not the case in other studies [6, 23, 32]. However, graft integrity [5, 22, 30, 32] and synovial coverage [22, 30, 32] had no correlation with stability in included studies.

**Table 4:** Summary of the studies that compare second-look findings with clinical outcomes

	Second-look findings	Clinical outcomes compared with second-look findings	Comments
Defrere et al. [19] (*CORR*, 1994)	Invested or non-invested (based on synovial coverage and neovascularization)	Stability (Lachman test, PST), failure	Invested (25): perfect stability (21), slight instability (4)
			Non-invested (22): perfect stability (13), slight instability (4), graft rupture in one half (1), Failure (4)
Toritsuka et al. [5] (*AOTS*, 2005)	Tension (probe test), integrity (tear)	KT-1000 (STSD)	1.1 mm (taut) v 2.3 mm (mildly lax/lax), *p* = 0.003
			1.2 mm (minimal tear) v 1.2 mm (substantial tear), *p* = 0.670
Ahn et al. [6] (*Arthroscopy*, 2007)	Tension (probe test)	KT-2000	No statistical difference in KT-2000 measurements between “good tension” and “some laxity” group
Ahn et al. [22] (*KSSTA*, 2007)	Tension (probe test), tear, synovial coverage, notch reformation, cyclops lesion	KT-2000 Lysholm score	“Normal tension” group was significantly better than “slightly lax” group in KT-2000, but there was no significant difference in Lysholm score between groups
			Graft tear, synovial coverage, notch reformation, and cyclops-like lesion showed no clinical correlation with KT-2000 and Lysholm score.
Kondo et al. [7] (*Arthroscopy*, 2007)	Three categories based on maturation score	Stability (KT-2000, PST) Clinical outcomes (ROM, Lysholm and IKDC score, mean isokinetic peak torque)	Category I showed significantly better results than categories II or III in KT-2000 and PST
			No significant differences between the three categories in all clinical outcomes
Otsubo et al. [23] (*KSSTA*, 2007)	Three groups based on tension (probe test) and integrity	KT-1000, PST	No significant differences between the three groups in KT-1000 and PST
Lee et al. [24] (*Arthroscopy*, 2010)	Synovial coverage, integrity, tension (probe test), cyclops or impingement	IKDC grade	Normal synovial coverage group and normal tension group had significantly more cases with IKDC grade A/B than the abnormal groups
			Integrity and impingement/cyclops made no difference in IKDC grade
Tanaka et al. [30] (*KSSTA*, 2012)	Graft damage and synovial coverage of PL	IKDC grade, Lachman test, PST, KT arthrometer	PL graft damage or synovial coverage showed no significant correlation with objective outcomes
Nakamae et al. [32] (*BJJ*, 2014)	Graft condition (integrity and tension), synovial coverage	KT-2000 and Lysholm score	Graft condition and extent of synovial coverage did not significantly affect the results of KT-2000 and Lysholm score

*PST* pivot shift test, *STSD* side to side difference, *ROM* range of motion

*CORR* Clinical Orthopaedics and Related Research, *AOTS* Archives of Orthopaedic and Trauma Surgery, *KSSTA* Knee Surgery, Sports Traumatology, Arthroscopy, *BJJ* The Bone & Joint Journal

On the other hand, only three studies investigated the correlation between second-look findings and patient-reported outcomes (IKDC subjective score, Lysholm score) [7, 22, 32]. All three of these studies reported that there was no correlation between second-look findings and patient-reported outcomes.

## Discussion

This systematic review summarizes how graft maturation was evaluated on second-look arthroscopy following ACL reconstruction. All included studies evaluated graft maturation with two or more of the following three gross findings: graft integrity, tension, and synovial coverage. Two to four categories were used for evaluating each parameter, but the criteria for classification were slightly different for each study. Several studies also used the parameter of neo-vascularization to evaluate the viability of grafts.

Follow-up period from ACL reconstruction to second-look arthroscopy varied according to the study, but the average length was about 1–2 years. Second-look arthroscopy to evaluate a graft should be performed only after the maturation of the graft ends. However, previous studies have reported different results on the timing of graft remodeling. Falconiero et al. [35] reported no significant differences in the histologic aspect of 12-month grafts evaluated by them. According to Sanchez et al. [36], the grafts reached maturity at around 2 years after surgery. Rougraff et al. [37] observed areas of degeneration, neo-vascularity, and hypercellularity until 3 years after reconstruction.

Graft integrity is the most basic evaluation method and almost all studies used integrity for evaluating graft maturation after ACL reconstruction. This was expressed as the presence and extent of the graft tear. In the majority of studies, graft integrity was classified into three categories: intact, partial (or superficial) tear, and complete (or substantial) tear. However, intact graft with no graft tears were mostly categorized as normal, whereas Ohsawa et al. classified normal integrity as over 80% [28, 29] and several studies followed this classification [10, 18]. The criteria for dividing the superficial tear and the substantial tear were also ambiguous, and some subjective judgment could be involved.

Tension of the graft is one of the most important evaluation parameters because it assesses the stability of the knee joint, which is the main purpose of ACL reconstruction. The tension of grafts was evaluated as taut, slightly lax, and lax by probing during second-look arthroscopy. This classification is problematic in that it depends on the subjective judgment of the examiner. Some studies described the use of rulers at the time of probing or the use of anatomical indicators such as femoral condyle to measure the tension of grafts, but this may also not be objective. In addition, the measurement of stability by arthroscopic probing has the disadvantage that it is difficult to evaluate the stability of rotation. Therefore, a method is needed that can be used to evaluate stability of rotation more objectively.

While the integrity and tension of grafts represent mechanical properties, synovial coverage has been used as a measure of biologic maturation. Synovial coverage of the graft was also mostly categorized into three categories, while the criteria for classification varied by study. Some studies regarded the best category as complete coverage [6, 7, 18, 28, 30], but a few other studies classified it as more than 80% [8, 10, 32, 33] or 50% [24] and some other studies as more than 20% [14]. This seems to be influenced by the subjective philosophy of each surgeon as to how much synovial coverage is considered good. In addition, the method used for measuring the amount of synovial coverage has not always been clearly described. Therefore, consensus and unification of evaluation criteria for synovial coverage are required.

Recently, long term follow-up studies of ACL reconstruction have been published, and long-term survival of grafts is recognized as important. Bottoni et al. [2] reported higher long-term failure rates for allografts compared to autografts. The high long-term survival rate of autografts is possibly because only well-matured grafts can survive the stresses incurred with a high level of activity in the long-term. With regard to this, it seems to be important to evaluate graft viability, such as the neo-vascularization of grafts, although only a few studies did this on second-look arthroscopy [9, 11, 18].

Several studies calculated the total maturation score by summing up the scores assigned to each evaluation parameter [7, 9–12, 17, 18]. Although these efforts have limitations that are not validated by the scoring system, it has the advantages of comparing graft maturation status between different graft types or surgical techniques or correlating graft maturation status with clinical outcomes. Kondo and Yasuda [7] reported that patients in the “excellent” group with higher total maturation score had better anteroposterior and rotational stability than those in the other groups. Ahn et al. [9] compared the anatomic reconstruction group with the non-anatomic reconstruction group and found that the former had better total graft maturation score and clinical outcomes. Kim et al. [11] demonstrated that total maturation scores of hamstring autografts were higher than those of tibialis allografts. In order to further develop such a total scoring system, efforts must be made to validate the results in comparison with clinical outcomes.

Although a few studies reported that graft tension in second-look arthroscopy was significantly correlated with objective stability test results [5, 22], second-look findings seem to be less correlated with clinical outcomes. Graft integrity [5, 22, 30, 32] and synovial coverage [22, 30, 32] had no correlation with stability in the included studies. Also, there was no correlation between second-look findings and patient-reported outcomes [7, 22, 32]. These results may be due to the subjective evaluation of second-look arthroscopy and the use of evaluation methods that have not yet been validated.

This study has several limitations. First, only five randomized trials, among 28 studies, were included for this systematic review. The quality of included studies was assessed to be relatively low (mean mCMS of 46.4 ± 6.7). Second, this study summarizes previous methods for evaluating graft maturation on second-look arthroscopy following ACL reconstruction, but it does not answer which evaluation method is appropriate. Consensus and validation are needed to determine which method is the most appropriate evaluation method. Third, the follow-up periods of the studies were relatively short and no long-term follow-up studies were included, and most studies had difficulty identifying the relationship between clinical outcomes and the evaluation of graft maturation. Finally, almost all the studies were from northeast Asia, including South Korea, Japan, and China; therefore, most studies are limited to specific races.

## Conclusions

Graft integrity, tension, and synovial coverage were the most frequently evaluated criteria for graft maturation on second-look arthroscopy following ACL reconstruction. However, there is no uniform criterion for evaluation of each parameter. Therefore, development of a validated evaluation method for second-look arthroscopy is required.

## Abbreviations

ACL: Anterior cruciate ligament
mCMS: Modified Coleman Methodology Scores
PRISMA: Preferred Reporting Items for Systematic Reviews and Meta-Analyses
